# Rare isolation of human-tropic recombinant porcine endogenous retroviruses PERV-A/C from Göttingen minipigs

**DOI:** 10.1186/s12985-022-01742-0

**Published:** 2022-02-21

**Authors:** Sabrina Halecker, Ludwig Krabben, Yannick Kristiansen, Luise Krüger, Lars Möller, Dietmar Becher, Michael Laue, Benedikt Kaufer, Christian Reimer, Joachim Denner

**Affiliations:** 1 Institute of Virology, Free University Berlin, Berlin, Germany; 2 Robert Koch Institute, Berlin, Germany; 3 Centre for Biological Threats and Special Pathogens ZBS 4: Advanced Light and Electron Microscopy, Robert Koch Institute, Berlin, Germany; 4 MICROMUN, Privates Institut Für Mikrobiologische Forschung GmbH, Greifswald, Germany; 5 Department of Animal Sciences, Animal Breeding and Genetics Group, University of Göttingen, Albrecht-Thaer-Weg 3, 37075 Göttingen, Germany; 6 Center for Integrated Breeding Research, University of Göttingen, Carl-Sprengel-Weg 1, 37075 Göttingen, Germany

**Keywords:** Xenotransplantation, Porcine endogenous retroviruses, Recombination, Long terminal repeats, Copy number

## Abstract

**Background:**

Porcine endogenous retroviruses (PERVs) can infect human cells and pose a risk for xenotransplantation when pig cells, tissues or organs are transplanted to human recipients. Xenotransplantation holds great promise to overcome the shortage of human donor organs after solving the problems of rejection, functionality and virus safety. We recently described the transmission of a human-tropic recombinant PERV-A/C, designated PERV-F, from peripheral blood mononuclear cells (PBMCs) of a Göttingen Minipig (GöMP) to human 293 cells (Krüger et al., in Viruses 12(1):38, 2019). The goal of this study was to characterize PERV-F in more detail and to analyze the probability of virus isolation from other animals.

**Methods:**

The recombination site in the envelope (*env*) gene, the long terminal repeats (LTR), the proteins and the morphology of the recombinant PERV-F were characterized by polymerase chain reaction (PCR), sequencing, Western blot analysis, immunofluorescence, and transmissible electron microscopy. Mitogen-stimulated PBMCs from 47 additional pigs, including 17 new GöMP, were co-cultured with highly susceptible human 293 T cells, and the PERV-A/C prevalence and PERV transmission was analyzed by PCR.

**Results:**

PERV-F, isolated from a GöMP, is an infectious human-tropic PERV-A/C virus with a novel type of recombination in the *env* gene. The length of the LTR of PERV-F increased after passaging on human cells. In a few minipigs, but not in German landrace pigs, PERV-A/C were found. There was no transmission of human-tropic PERV-A/C from additional 47 pigs, including 17 GöMP, to human cells.

**Conclusion:**

These data show that human-tropic recombinant PERV-A/C proviruses can only be found in a very small number of minipigs, but not in other pigs, and that their isolation as infectious virus able to replicate on human cells is an extremely rare event, even when using highly susceptible 293 cells.

**Supplementary Information:**

The online version contains supplementary material available at 10.1186/s12985-022-01742-0.

## Background

Porcine endogenous retroviruses (PERVs) are gamma retroviruses integrated in the genome of all pigs. There are three different PERV subtypes: PERV-A, PERV-B and PERV-C [1]. Whereas PERV-A and PERV-B are integrated in the genome of all pigs, PERV-C is found in many, but not all pigs. PERV-A and PERV-B infect cells from numerous species including human cells [1–3]. The human porcine endogenous retrovirus-A receptor 1 and 2 (huPAR1 and huPAR2, respectively) are receptors for PERV-A [4]. They are members of the riboflavin transporter, also known as human riboflavin transporter 3 (hRFT3), and human riboflavin transporter 1 (hRFT1), respectively. More recently, these receptors have been renamed and classified as members of the solute carrier family of receptors, “solute carrier family 52A” (SLC52A) [5]. huPAR1 and huPAR2 are present on human cells, related receptors are present on other species, determining the host range. The PERV receptor on baboon and other non-human primate cells is functional, but deficient by a mutation, explaining the low replication in these cells [6]. Mice have a mutated receptor, explaining that mouse cells could not be infected, and rats have only a low expression of a functional receptor, explaining that rat cells could not be infected [7]. However, transfection with human or rat PAR-1 conferred susceptibility [7]. The receptors for PERV-B and PERV-C are still unknown. PERV-C is in contrast to PERV-A and PERV-B an ecotropic virus, infecting only pig cells [8]. The envelope proteins of the viruses are responsible for infection. The receptor-binding domain of the surface envelope protein interacts with the cellular receptor and the transmembrane envelope protein is responsible for the fusion between viral and cellular membranes [1]. In some pigs recombinants between PERV-A and PERV-C were found [9]. The recombinant viruses contain the receptor-binding domain of PERV-A and most of the PERV-C genome, including the PERV-C-specific long terminal repeats (LTRs). The recombination point in the envelope gene (*env*) is well known, however the second recombination point, which should be upstream of the *env* gene is not known. PERV-A/C viruses are replication competent in living pigs and numerous copies were found in different organs of these pigs, but never in the germ line. PERV-C-positive miniature pigs from the USA have been shown to harbour such PERV-A/C viruses [9, 10] and their mitogen-stimulated PBMCs released human-tropic viruses able to infect 293 cells [11]. PERV-A/Cs were also found in Yucatan micropigs and their PBMCs were characterised by a high expression of PERV and release of reverse transcriptase (RT) activity into the supernatant [11–13]. Until now PERV-A/Cs were described with one exception only in minipigs, possibly as the result of inbreeding, higher numbers and higher expression of PERV-C in these animals (for review see [9]). In a previous publication we analysed GöMPs produced at Ellegaard Göttingen Minipigs A/S (Dalmose, Denmark) and did not find any PERV-A/C in the PBMCs from five animals [14]. Recently, we screened Göttingen minipigs produced at the University of Göttingen (Göttingen, Germany) for the presence of human-tropic PERVs and found that mitogen-stimulated PBMCs from one of 11 analyzed animals, named pig F, were able to infect human 293 cells, either by the release of cell-free virus or by cell-to-cell transmission [15]. One of these mechanisms is a prerequisite for the infection of recipients after xenotransplantation. However, this is a rare event: In our experiment, PBMCs from one of 11 Göttingen minipigs from the University of Göttingen were able to infect human 293 cells, either directly or cell-free by released virus [15]. Here, we give an extended analysis of this virus including changes in the long terminals repeats (LTRs) during passaging on human cells. In order to evaluate the probability of the virus release, the experiment was performed with other pigs, including new GöMPs.

## Methods

### Animals

The German Landrace pigs were from the Institute for Molecular Animal Breeding and Biotechnology, Faculty of Veterinary Medicine, Ludwig-Maximilians-University ((LMU), Munich, Germany) [16], the Black Forest pigs from the Molecular Animal Breeding and Biotechnology, Gene Center, LMU (Oberschleißheim, Germany) [17] and the Aachen minipigs from the Aachen minipigs (Heinsberg, Germany) [18] (Table 1). The history of GöMP and the conditions of breeding at the University of Göttingen were described in detail in an earlier study [15]. In the first experiment, 11 GöMP from the University of Göttingen [15] and 19 Landrace, 5 Black Forest and 6 Aachen minipigs were analyzed. In the second experiment, blood from additional 17 GöMP was obtained from the University of Göttingen, 12 of the animals were half siblings on the maternal side, one of them half siblings on the paternal side of pig F, which released a human-tropic PERV-A/C [15].

**Table 1:** Animals tested

Pig breeds	Number of tested animals	Detection of			Animals positive for PERV-A/C	Infection of 293 cells
		PERV-C	PERV-A/C^a^	PERV-A/C^b^		
Göttingen minipigs^c^	11	11	3	3	5	1
Göttingen minipigs^d^	17	17	1	0	1	0
German landrace^c^	19	19	0	0	0	0
Black forest minipig^c^	5	5	1	0	1	0
Aachen minipig^c^	6	6	0	2	2	0
In total	58	58 (100%)	5 (8.6%)	5 (8.6%)	9 (15.5%)	1 (1.7%)

^a^PCR using the primer pair PERV-A VRB fw and PERV-C TMR rev (1200 bp amplicon),

^b^PCR using the primer pair PERV-A VRB fw and PERV-C rev (380 bp amplicon),

^c^Experiment 1 [15] and this manuscript,

^d^Experiment 2, this manuscript

### Cell culture

Human embryonic kidney 293 cells (HEK293 or 293 cells) were cultured in DMEM supplemented with 10% FCS, 2 mM L-glutamine, 50 units penicillin and streptomycin. Pig PBMCs were cultured in RPMI 1640 supplemented with 10% FCS, 2 mM L-glutamine, 50 units penicillin and streptomycin or stored frozen in liquid nitrogen [15]. For the co-cultivation experiment 6 × 10^4^ 293 cells were seeded in a well of a 12-well plate and 24 h later 1 × 10^6^ PBMCs from 11 GöMP and 30 other pigs (Table 1) stimulated for five days with 9.6 µg/mL phytohemagglutinin (PHA-L, Biochrom, Berlin, Gemany) were added. The 293 cells-PBMC co-cultures were split when 90–95% confluency was reached. For cell-free infections, sterile-filtered supernatant from PBMCs of animal F or PERV-producing 293 cells was added to uninfected 293 cells after washing with PBS. In the second co-cultivation experiment 1 × 10^6^ unstimulated PBMCs from 17 GöMP were added directly to 50% confluent 293 cells with 10 µg/ml PHA-L (Invitrogen, Whaltham, MA). Cells were incubated for five days before splitting.

### DNA and RNA isolation

In the first experiment, involving 11 GöMP and 30 other pigs (Table 1), DNA was extracted from PBMCs or 293 cells with two extraction kits: DNeasy Blood and Tissue kit (Qiagen GmbH, Hilden, Germany) and NucleoSpin Virus (Macherey–Nagel, Düren, Germany). DNA was quantified and the 260 nm/280 nm ratio was determined using a NanoDrop ND-1000 (Thermo Fisher Scientific Inc., Worcester, MA, USA). RNA was extracted from blood or cells with RNeasy Mini kit (Qiagen GmbH, Hilden, Germany) following the manufacturer´s instructions [15]. In the second experiment, involving 17 GöMPs, DNA extraction of the harvested cell suspensions containing 293 T cells and residual PBMCs were carried out with innuPREP Virus DNA/ RNA Kit (Analytik Jena, Jena, Germany) according to the manufacturer´s instructions. RNA was extracted from cell suspensions obtained by the incubation of 293 T cells with PBMCs of pig 8. DNA was digested using the RNase-free DNase Set (Qiagen, Hilden, Germany) and RNA extracted with RNeasy Mini kit (Qiagen, Hilden, Germany).

### Polymerase chain reaction (PCR) and droplet digital PCR (ddPCR)

PCR methods or reverse transcriptase PCR (RT-PCR) methods were performed using specific primers (Table 2). Using a primer pair located in the pol region of PERV (PERVpol) all PERV subtypes were detected [19], using specific primers binding to the *env* genes, PERV-C and PERV-A/C were detected [13, 20]. Reverse transcribed DNase treated RNA was used with PERVpol specific primers to detect the full-length mRNA and expression of PERV. The full-length mRNA encodes the groups-specific antigen (Gag) and polymerase (Pol) proteins. Finally, a real-time PCR using primers and probes specific for porcine GAPDH (pGAPDH) and porcine actin was applied for normalization (Table 2).

**Table 2:** Primers and probes used for the detection and characterisation of PERV

PCR assay	Primer/probe	Sequence 5′–3′	Accession number	Position	References
*Primers and probes for ddPCR*					
PERV pol	PERV pol—forward	CGA CTG CCC CAA GGG TTC AA	HM159246	3568–3587	Yang et al. [31]
	PERV pol—reverse	TCT CTC CTG CAA ATC TGG GCC		3803–3783	
	PERV pol—probe	6FAM-CAC GTA CTG GAG GAG GGT CAC CTG-BHQ1		3678–3655	
pGAPDH	pGAPDH—forward	TTC ACT CCG ACC TTC ACC AT	396823	3951–3970	
	pGAPDH—reverse	CCG CGA TCT AAT GTT CTC TTT C		4022–4001	
	pGAPDH—probe	HEX-CAG CCG CGT CCC TGA GAC AC-BHQ1		3991–3972	
pActin	pActin—forward	TAA CCG ATC CTT TCA AGC ATT T			
	pActin—reverse	TGG TTT CAA AGC TTG CAT CAT A			
	pActin—probe	HEX-CGT GGG GAT GCT TCC TGA GAA AG-BHQ1			
*Primers to detect PERV-C*					
PERV-C	PERV EnvC—forward	CCC CAA CCC AAG GAC CAG	AM229312	9601–9618	Kaulitz et al. [46]
	PERV EnvC—reverse	AAG TTT TGC CCC CAT TTT AGT		9692–9672	
	PERV EnvC—probe	6FAM-CTC TAA CAT AAC TTC TGG ATC AGA CCC-BHQ1		9626–9652	
*Primers to detect PERV-A/C*					
PERV-A/C (380 bp amplicon)	PERV-A-VRB—forward	CCT ACC AGT TAT AAT CAA TTT AAT TAT GGC	AY570980.1	6129–6158	Dieckhoff et al. [14]
	PERV-C reverse	TAT GTT AGA GGA TGG TCC TGG TC		6451–6473	
PERV-A/C (1200 bp amplicon)	PERV-A-VRB—forward	CCT ACC AGT TAT AAT CAA TTT AAT TAT GGC	AY570980.1	6129–6158	Wood et al. [10]
	PERV-C-TMR	CTC AAA CCA CCC TTG AGT AGT TTC C		7370–7395	
*Primers for LTR amplification*					
PERV LTR	PK34	AAA GGA TGA AAA TGC AAC CTA ACC	Y17012	3–26	Czauderna et al. [45]
	PK26	ACG CAC AAG ACA AAG ACA CAC GAA		1134-1111	

Droplet digital PCR was done according to the manufacturer’s instructions (Bio-Rad, Hercules, CA, USA [http://www.bio-rad.com/de-de/applications-technologies/droplet-digital-pcr-ddpcr-technology?ID=MDV31M4VY]) using a QX200 droplet generator and a QX100 droplet reader (Bio-Rad). Purified genomic DNA from cultured cells (50 ng genomic DNA) was digested with MseI (New England Biolabs, USA) (20U) at 37 °C for 1 h. The ddPCR mix consisted of 10 μL 2 × ddPCR Master mix, 1.8 μL of each 10 µM target primers (Table 2), 0.5 µL of each 10 µM probes (FAM/HEX) (Table 2). The DNA digest had a concentration of 2.5 ng/µL and 2 µL corresponding to 5 ng digested DNA were added to the mix. Water was added to a total volume of 20 μL. PCR cycling conditions: 10 min initial enzyme activation at 95 °C, 30 s denaturation at 94 °C, 1 min annealing and extension at 60 °C (40 cycles) and final 10 min enzyme deactivation at 98 °C using a Master cycler ProS (Eppendorf). The temperature ramp rate was 2 °C per second. Porcine actin was used as reference (Table 2).

### Transmission electron microscopy

PERV-F producing 293 T cells were fixed with 2.5% glutaraldehyde in 50 mM 2-[4-(2-hydroxyethyl)piperazin-1-yl]ethanesulfonic acid (HEPES), pH 7.2. The cells were harvested by scraping, pelleted at 2.000 × g for 5 min at 4 °C, and washed twice with HEPES. After washing, the cells were block-embedded by mixing equal amounts of pelleted cells and low-melting-agarose (3%). Agarose-embedded cells were cut into small pieces (< 1 mm), and postfixed with osmium tetroxide (1% in double distilled H_2_O for 1 h), tannic acid (0.1% in 50 mM HEPES for 30 min), and uranyl acetate (2% in ddH_2_O for 2 h). The agarose-embedded cells were dehydrated in a graduated ethanol series and finally embedded in Epon resin. Thin sections (60–70 nm) were cut with a Leica UC7 ultramicrotome, using a diamond knife (45°, Diatome, Switzerland), mounted on naked 300 mesh copper grids, and counterstained with uranyl acetate (2% in ddH_2_O for 20 min), followed by lead citrate (Reynolds’ solution for 3 min). Ultrathin sections were stabilized with a thin layer of carbon evaporation and examined using a JEM-2100 transmission electron microscope (JEOL) at 200 kV. Images were recorded using a Veleta CCD camera (EMSIS) and 2048 × 2048 pixel.

### Sequencing

Amplicons obtained after a specific PERV-A/C PCR using the pimers PERV-A VRB Foreward (fw) and PERV-C TMR reverse (rev) (Table 2) were purified and either directly sent for sequencing (LGC Genomics, Berlin, Germany) or cloned into a TOPO A vector (ThermoFisher Scientific) and sent for sequencing. Plasmid preparation from single colonies were sequenced.

### Immmunofluorescence

Goat antiserum 30 against the p27Gag protein of PERV [21], which had been used previously in Western blot, immunofluorescence, and immunoperoxidase assay (IPA) [22], as well as immunohistochemistry [23] analyses, was used to detect protein expression. PERV-F infected 293 T cells in 96 well microtitre plates were washed, fixed with cold aceton-methanol, blocked with 2% fat-free milk solution, incubated with 1:100 anti-p27Gag serum and 1:300 rabbit anti-goat FITC-labelled serum.

### Western blot analysis

Lysates from 293 cells infected with PERV-F [15] and from 293 cells infected with PERVi, were prepared. PERVi was obtained by infecting uninfected 293 T cells with cell-free supernatant of PERV-5°, a virus well characterized [24, 25]. In addition, uninfected 293 cells were used as negative control and the recombinant surface envelope protein gp70 and the core p27Gag protein as positive control. Virus pellets were obtained by ultracentrifugation of cell-free supernatant from PERV producing 293 cells. The cell lysates were analysed directly, and in 1:10 and 1:100 dilutions with goat serum 62 to detect the surface envelope protein gp70, goat serum 30 to detect the core protein p27Gag and goat serum 16 to detect the transmembrane envelope protein p15E. All sera were used 1:1000, an alkaline phosphatase conjugates donkey anti-goat serum was used 1:2000. Dilutions of cell lysates or virus pellets were dissolved in sample buffer (50 mM Tris–HCl, 12% glycerol, 4% SDS, 5% β- mercaptoethanol, 0.01% bromophenol blue) and denatured for 5 min at 95 °C prior to electrophoresis. Proteins were separated on a 10% polyacrylamide gel together with either unstained or pre-stained protein ladders (both PageRuler, ThermoFisher, Waltham, USA). Samples were transferred for 50 min to nitrocellulose membranes by semi-dry blotting (15 V), stained with Ponceau red, cut into strips and blocked over night at 4 °C with 5% blotting grade dry milk (Carl Roth, Karlsruhe, Germany) in PBS with 0.05% Tween 20 (blocking buffer). Strips were incubated with sera diluted 1:300 in blocking buffer for 2 h at room temperature. Staining was performed with 3,3′-diaminobenzidine (DAB) (Thermo Fisher) or with 5-bromo-4-chloro-3-indolyl-phosphate nitro blue tetrazolium (NBT/BCI) (Promega, Madison, USA).

## Results

### Screening different pig strains for PERV-A/C

**Fig. 1:**
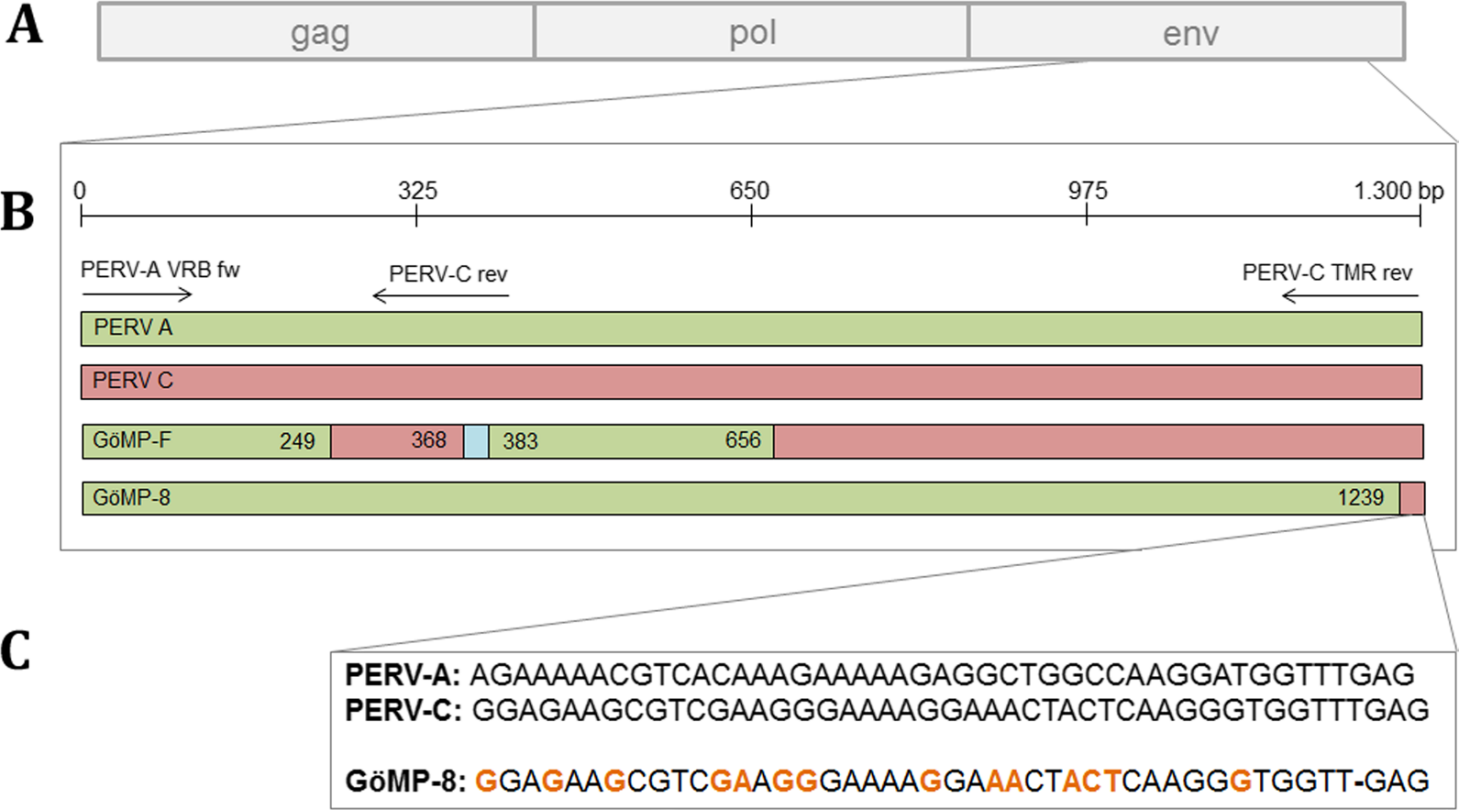
Schematic presentation of the recombination sites in the envelope gene of PERV A/C isolates. **A** genomic organization of PERVs, **B** recombination points of various PERV A/C isolates, GöMP-F, virus released from the Göttingen Minipig F, GöMP-8, virus released from the Göttingen minipig number 8. Numbers indicate the localization of the recombination site in base pairs (bp), **C** comparison of a short sequence in the env gene of PERV-A (Accession No. AJ293656), PERV-C (Accession No. KY352351) and PERV-A/C isolated from GöMP 8. Green indicate PERV-A, red PERV-C, blue is a 15 nt insert in PERV-F, the numbers indicate the last nt of the recombination point

**Fig. 2:**
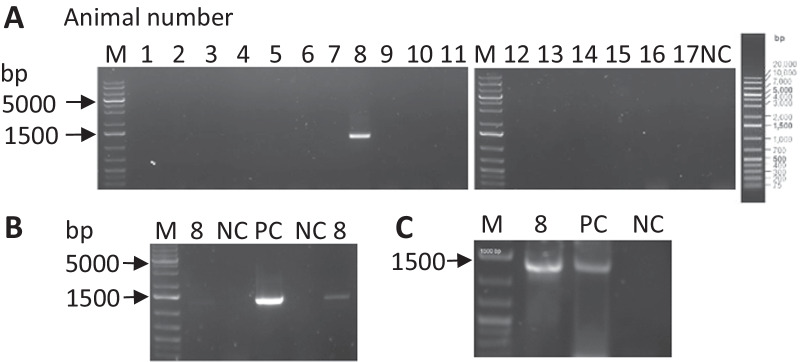
**A** Detection of a PERV-A/C provirus in untreated PBMCs from GöMP 8 and absence of PERV-A/C in the PBMCs of 16 other GöMPs. NC, negative water control. The migration of the markers is also shown. **B** Confirmation of the detection of an PERV-A/C provirus in untreated PBMCs from pig number 8, NC, negative water control, PC, positive control, M marker. **C** Detection of a PERV-A/C mRNA using primers PERV-A VRB fw and PERV-C TMR rev in DNase-treated RNA from PBMCs of pig 8, PC positive control, NC negative control

PBMCs from 58 different pigs were screened by PCR for PERV-C, a prerequisite for the generation of PERV-A/C. All animals were found positive for the PERV subtype C (Table 1). For the detection of PERV-A/C two primer pairs were used. The primer pair PERV-A VRB fw and PERV-C TMR rev (1200 bp amplicon) were used for the detection of a longer region in env (Fig. 1). This longer region was detected in the PBMCs from three GöMP from the University of Göttingen in the previous experiment [15], and in the PBMCs from one Black Forest minipig (Table 1). The other primer pair PERV-A VRB fw and PERV-C rev (350 bp amplicon) (Fig. 1) reacted positive with the PBMCs from three GöMPs and two Aachen minipigs (Table 1). In addition, the pig PBMCs were screened for human-tropic, replication competent PERV-A/C able to infect 293 cells. In the previous experiment [15], purified PBMCs from 11 GöMPs were stimulated five days with the mitogen PHA-L and then added to human 293 cells. The co-culture was repeatedly passaged, only PBMCs from one animal, named pig F, were able to infect human 293 cells. The virus was isolated and partially characterized [15]. None of the other animals was able to infect human 293 cells. Human 293 cells lack intracellular restriction factors, therefore they are highly susceptible to infection with human-tropic PERV [26]. It is well known that in contrast to these cells human primary cells cannot be infected easily [27]. In the second experiment, freshly isolated PBMCs from 17 other GöMP were screened for PERV-A/C using both primer pairs. When the primer pair PERV-A VRB fw and PERV-C TMR rev (1200 bp amplicon) was used, only in the PBMCs of one animal, pig 8, the corresponding amplicon was found (Fig. 2A, B). When the primers PERV-A VRB fw and PERV-C rev (350 bp amplicon) were used, no positive PCR-reaction was observed with DNA of animal 8 (not shown), indicating that the recombination took place upstream from the PERV-C rev primer binding site. Pig 8 was a female half sibling on the maternal side to animal F, which produced infectious PERV-A/C in the first experiment. In contrast to the previous experiment [15], the PBMCs of new 17 GöMP were stimulated with PHA-L and co-cultured with 293 cells at the same time. None of the co-cultures contained PERV-A/C sequences in the third passage (day 19 after co-cultivation). At the same time the test for PERV pol was positive, indicating that there were still pig PBMCs or pig DNA present in the culture of the passaged 293 cells. The test for PERV pol was performed as real-time PCR, the cycle threshold (Ct) values ranged from 22.6 to 28.4 (not shown). This means that in the case of animal 8 the small number of cells carrying PERV-A/C provirus at the beginning died after cultivation. However, it remains unclear, whether the provirus present at the beginning in the freshly isolated PBMCs of animal 8 did produce virus particles able to infect human 293 cells. At least viral RNA was detected in the PBMCs of animal 8, using env-specific primers (Fig. 2C).

To summarize, 58 pigs were screened of which 9 animals were tested positive for PERV-A/C and only one GöMP released infectious, human-tropic PERV-A/C. All other 57 animals did not release a PERV-A/C.

### Extended characterization of PERV-F

**Fig. 3:**
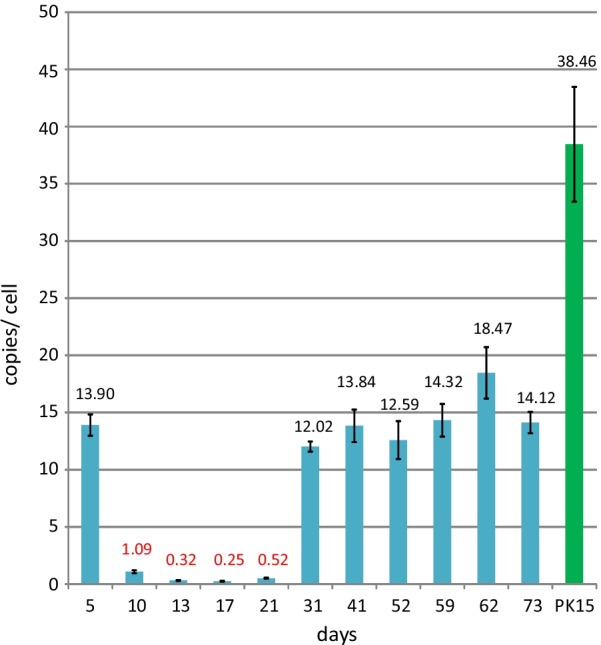
PERV copy numbers in consecutive passages of human 293 cells after co-incubation of stimulated PBMCs from pig F with PHA-L. The copy number was estimated by ddPCR using porcine actin as reference gene. For comparison the PERV copy number in pig PK15 cells was analysed (green column)

PERV-A/C from pig F was characterized in more detail by analysis of the co-culture of PBMCs from pig F with 293 cells. During the first passages of the co-culture the primary pig PBMCs were lost and only the immortalised human 293 cells survived. After each splitting, the copy number of PERV proviruses was measured by ddPCR (Fig. 3). On day 5, the PERV copy number was 14 copies per cell, obviously still due to the presence of pig PBMCs or pig DNA in the co-culture. During the next passages the pig PBMCs died and the low copy numbers measured (0.32–0.52 copies/cell) indicated that only a very low number of human 293 cells was infected. Beginning with day 31 the copy number increased, reaching a plateau at 14–18 PERV copies per cell. This increase indicated the presence of a replication-competent virus. The copy number at the end of the experiment was below the copy number in the original PBMCs from pig F (77.91 copies per cell), estimated also by ddPCR [15], and below the copy number of PERV-releasing PK15 cells which served as a standard (42 copies) (Fig. 3). This result confirms that PBMCs of animal F transmitted a replication-capable virus to human 293 cells. The virus could be transmitted cell-free from 293 cell culture supernatant to 293 cells (not shown).

### Gene expression of PERV

**Fig. 4:**
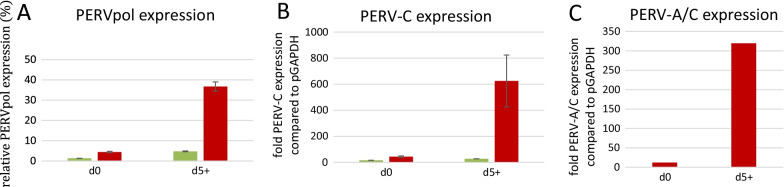
PERV expression in PBMCs from GöMPs F and E. **A** expression of PERV pol (shows the expression of all PERVs) of unstimulated PBMCs at day 0 (d0) and at day 5 after PHA stimulation (d5+) from pig F (red) compared to pig E (green). **B** Expression of PERV-C of unstimulated PBMCs at day 0 (d0) and at day 5 after PHA stimulation (d5+) from pig F (red) and for comparison from pig E (green). **C** Expression of PERV-A/C of unstimulated PBMCs at day 0 (d0) and at day 5 after PHA stimulation (d5+) from pig F (red). Error bars indicate standard error of mean, n = 3

When in the first experiment the expression of PERV in PBMCs from pig F was analysed by an RT-PCR using PERV pol primers, which detect all types of PERV, a strong increase in PERV expression was detected after mitogen stimulation at day 5 (Fig. 4A) [11]. These stimulated PBMCs had been used for the co-cultivation with human 293 cells described above. The expression of PERV in PBMCs from another animal, pig E, was much lower (Fig. 4A). The same difference in expression was found for the expression of PERV-C (Fig. 4B). Whereas in animal E no PERV-A/C was found, in the PBMCs of animal F PERV-A/C was found and its expression increased considerably after mitogen stimulation (Fig. 4C). This shows that RNA of PERV-A/C were present in the cultured PBMCs of animal F before added to the 293 cells.

### Passaging of PERV-F on human cells leads to changes in the LTR

**Fig. 5:**
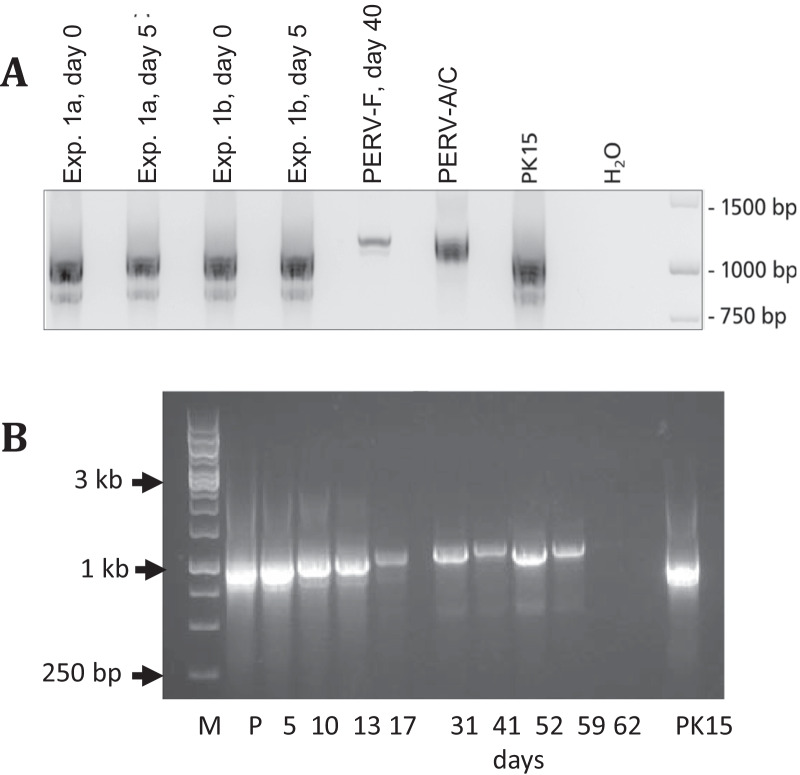
Amplification of the LTR sequences from DNA from different passages of human 293 cells infected with PERV-F. The days after start of the co-cultivation of uninfected 293 cells with mitogen-stimulated PBMCs from pig F are indicated. DNA was isolated from the cells and for amplification the primers AAAGGATGAAAATGCAACCTAACC and ACGCACAAGACAAAGACACACGAA (Czauderna et al., 2000 [45]) were used. **A** Results of two independent experiments on day 0, day 5 and day 40. PERV-A/C means the positive control of PERV-5^0^ [24, 25], PK15 was also used as control, **B** comparison of subsequent passages of PERV-F on 293 cells, M, marker, the Gene ruler 1 kb DNA ladder was used; P, DNA isolated from original PBMCs from pig F; PK15, DNA isolated from PK15 cells

**Fig. 6:**
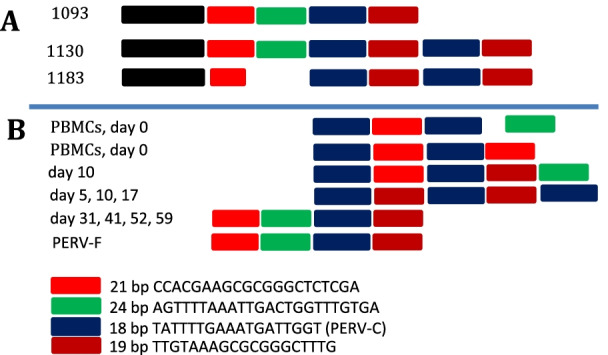
Schematic presentation of the repeats in the LTR of PERV-A/C. **A** Cell-free PERV-A/C was quickly passaged on human 293, leading to additional repeats, Denner et al., [24]. **B** Schematic presentation of the localization and number of repeats in LTRs isolated at different time points of co-culture of PBMCs from GöMP 8 with human 293 cells and subsequent passaging. PERV-F represents the LTR of a provirus after long-time passaging of PERV-F on 2893 cells

After repeated quick passaging of the PERV-F isolate on 293 cells, an increase in the length of the LTRs (Fig. 5) was observed using specific primers for the LTR sequences (Table 2). Previously published work demonstrated that the length of the LTR increased after repeated quick passaging of an PERV-A/C isolated from a NIH minipig on human 293 cells [24, 28]. This increase was due to the multimerization of repeats, which represent transcription factor binding sites. To analyse whether the same happened with PERV-F, we sequenced the LTRs after each passage either using the amplicon or cloned LTR sequences. Different arrangements of repeats were found, which were in some cases different to the arrangements of the repeats observed previously (Fig. 6A). In some sequences the following repeats were found: 18 bp (TATTTTGAAATGATTGGT)-21 bp (CCACGAAGCGCGGGCTCTCGA)-18 bp-21 bp-18 bp (Fig. 6B). These sequences are in agreement with longer LTR sequences. However, we were unable to sequence a complete long-sequence LTR and after further cultivation we lost the virus with the long LTR. At that time, LTRs with the sequence order 21 bp–24 bp (AGTTTTAAATTGACTGGTTTGTGA)-18 bp-19 bp (TTGTAAAGCGCGGGCTTTG) were found (Fig. 6B). To verify the results of the first experiment, we started a second experiment with 17 other GöMP, which were related to the animal F, that released a PERV-A/C isolate in the first experiment. However, since no replication competent virus was detected in the second experiment, we were unable to repeat the study of changes in the LTR.

### Determination of the recombination sites in the envelope sequence

PERV-F is a recombinant PERV-A/C virus with a recombination in the env sequence of PERV-C acquiring the receptor binding site of PERV-A in the backbone of PERV-C. As reported previously, PERV-F has two recombination sites [15] (Fig. 1).

The analysis of the recombination point in the PERV-A/C provirus from animal 8 in the second experiment showed no result for the 380 bp amplicon (primer pair PERV-A VRB fw and PERV-C rev, Fig. 1), but only the 1200 bp amplicon (primer pair PERV-A VRB fw and PERV-C TMR rev) was detected. Hence, the recombination point is upstream the binding site of PERV-C rev primer (Fig. 1). This was confirmed by cloning and sequencing the amplicon (Fig. 1; Additional file 1: Fig. 1). When the recombination site in PERV-F was compared with the recombination sites of other recombinant PERV-A/C viruses such as PERV-5^0^, which was obtained after passaging PERV-3^0^-NIH on human 293 cells [24], recombinant PERV-A 14/220 [29] and other published PERV-A/C sequences [19], differences in the break points were observed (Fig. 1, see also [9]). These data indicate that all recombinations were independent events.

### Protein production and virus release

**Fig. 7:**
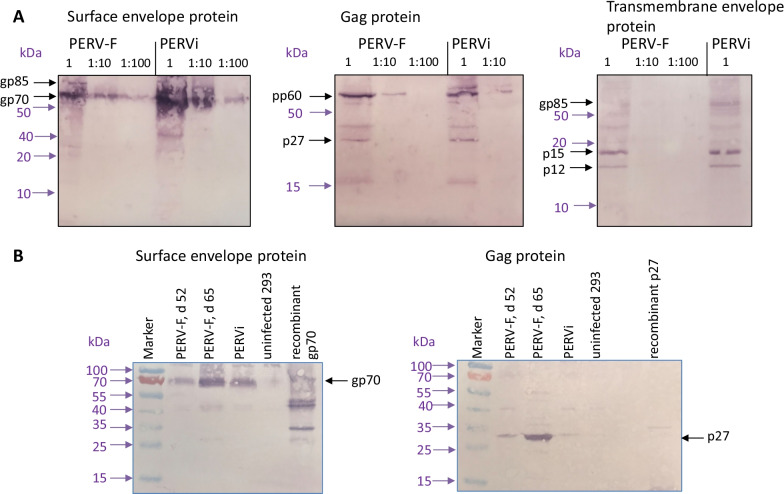
Detection of viral proteins by Western blot analysis. **A** Cell lysates from 293 cells infected with PERV-F and with PERVi were analysed in 1, 1:10 and 1:100 dilution, using goat serum 62 to detect the SU-Env gp70, goat serum 30 to detect the p27Gag and goat serum 16 to detect the transmembrane envelope protein p15E. PERVi is a new infection (i) of PERV-5^0^ [24]. The sera were used 1:1000, an alkaline phosphatase conjugates donkey anti-goat serum was used 1:2000. An unstained protein ladder was used and the molecular weights in kDa were indicated with lilac arrows. **B** Virus pellets from supernatants of 293 cells producing PERV-F at day 52 and 65 and 293 cells producing PERVi were analysed using goat serum 62 and 30, recombinant gp70 (molecular weight 54) and recombinant p27 were used as positive control. A prestained protein ladder was used and the molecular weights in kDa were indicated with lilac arrows

**Fig. 8:**
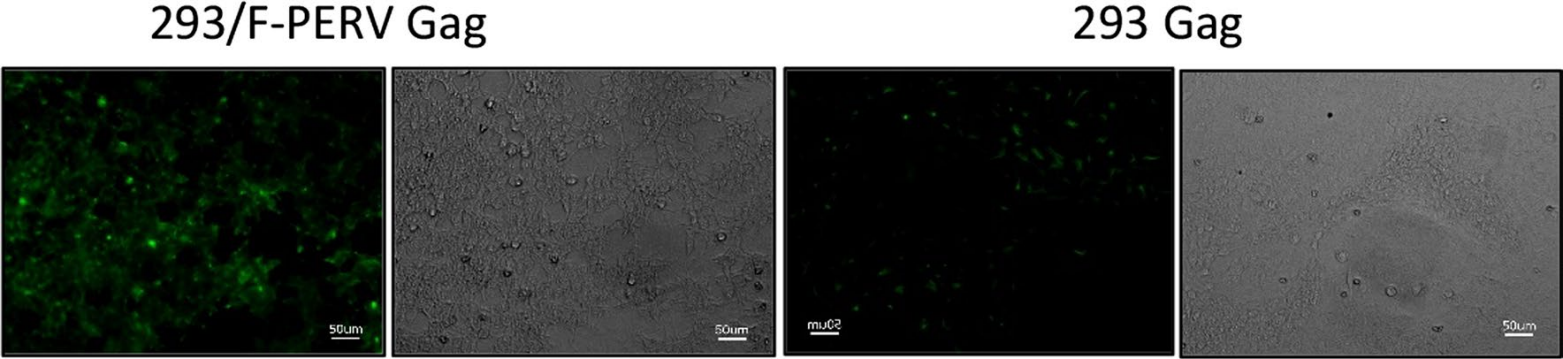
Immunofluorescence analysis of PERV protein expression in 293 cells infected with PERV-F. An antiserum against p27Gag was used. As control uninfected 293 were analysed

293 cells producing PERV-F were analysed by Western blot analysis using specific antibodies against three PERV proteins, p15E, p27Gag and gp70SU Env. All three proteins were detected in cell lysates as well as in pelleted virus particles from the supernatant (Fig. 7). In addition to gp70 and p27 the precursor molecules gp85 and pp60, respectively, were found. Next to p15E the p12 after cleavage of the R peptide was seen in the cell extracts. The expression of PERV proteins was confirmed by immunofluorescence analysis (Fig. 8). The release of infectious virus was shown by cell-free transmission of the infection to uninfected 293 cells (not shown).

### Additional morphological characterisation

**Fig. 9:**
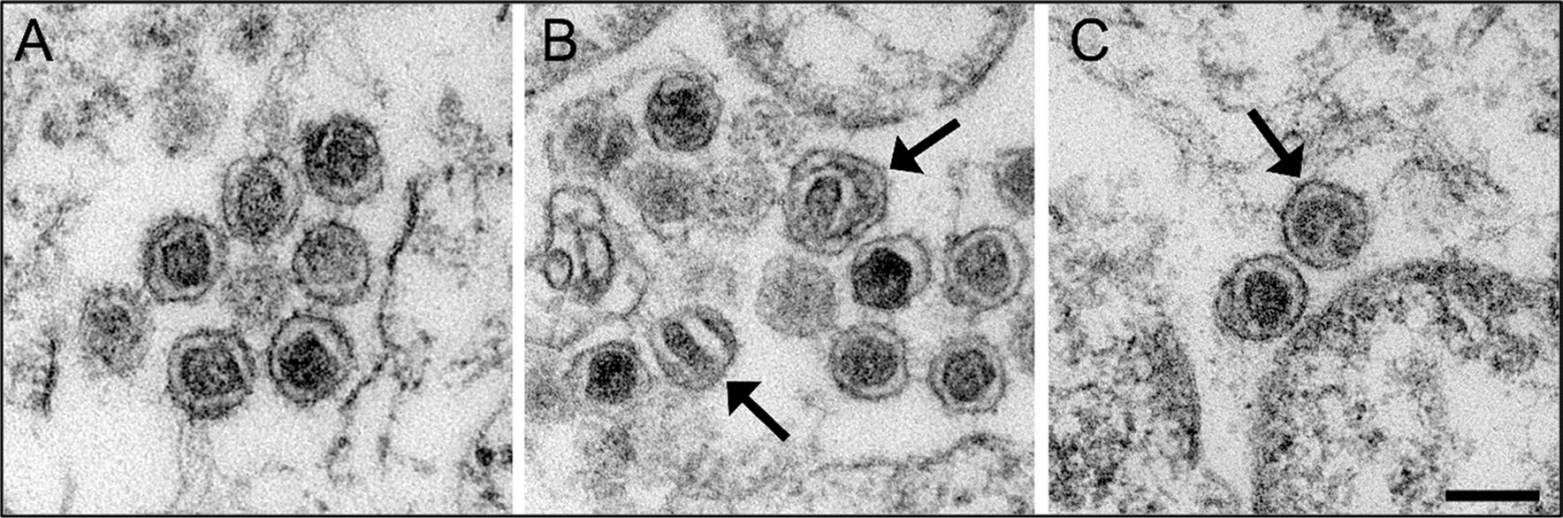
Electron microscopy of PERV particles produced in 293 cells. **A** group of mature virions with icosahedral nucleocapsids (C-type morphology), **B**, **C** Groups of viruses with few particles showing an atypical nucleocapsid morphology (arrows) which is clearly different from the icosahedral shape of mature virions but also from the ring-like gag-protein arrangement of immature virus particles. Scale bar = 100 nm

An extended analysis of the morphology of PERV-F particles that were released after co-cultivation of 293 cells with porcine PBMCs by thin section electron microscopy was performed. Most of the virus particles revealed a C-type morphology, i.e. a retrovirus particle with an icosahedral nucleocapsid, as described previously [15]. Besides particles with the typical C-type morphology, atypical particles with divergent nucleocapsid morphology were detected by a more extended analysis (Fig. 9). The occasional presence of particles with divergent nucleocapsid or virus morphology in a population of morphologically well-defined virus particles is rather common in retroviruses and other enveloped viruses, but is only rarely documented [30]. The virus particles with a divergent nucleocapsid structure detected here were different from PERV particles which were released from PK15 cells after treatment with a PERV-specific CRISP/Cas in order to inactivate the reverse transcriptase of PERV [31, 32]. The particles produced after applying this procedure were immature and showed ring or horseshoe-like nucleocapsids. An altered expression of the core protein Gag in the CRISPR-Cas treated PK15 cells [32] suggests an off-target effect on the Gag protein or the protease which might prevent nucleocapsid maturation.

## Discussion

Analysing the ability of PBMCs from 58 different pigs to produce a human-tropic PERV, able to infect human 293 cells, we found that only one pig, a GöMP, was able to do so, either by the release of an infectious virus or by cell-cell-contact. This indicates that the probability of a human-tropic PERV transmission is very low. The fact that a PERV-A/C was isolated confirms previous reports showing that the viruses isolated by co-culture of pig PBMCs with human cells usually were recombinant PERV-A/Cs [9, 15, 33]. These recombinant viruses acquired the receptor binding domain of PERV-A on the backbone of PERV-C, which allows them to infect human cells [10, 11, 15]. Therefore, the elimination of PERV-C is of great importance for a safe xenotransplantation, because a recombination with PERV-A cannot occur in the absence of PERV-C. Recombinant PERV-A/C are characterised by a high proliferation rate and an isoleucine to valine substitution at position 140 in the receptor binding domain and mutations in the proline rich region of the envelope were found [25, 34]. However, this recombination is not the only way how PERV-C may change to be able to infect human cells: mutations in the proline rich region of the envelope protein also allowed PERV-C to gain human tropism [20].

As described, quick passaging of a PERV-A/C isolate on human 293 cells was associated with a higher replication rate [24, 35]. The higher replication capacity of recombinant PERV-A/C may be associated with a higher infectivity and pathogenicity as it was shown in the case of other retroviruses, e.g., human immunodeficiency virus (HIV) [36], simian immunodeficiency virus (SIV) [37] and feline leukaemia virus (FeLV) [38]. Higher virus loads of human T-lymphotropic virus increase the transmissibility of the virus [39].

Passaging of a PERV-A/C virus on human 293 cells under selection pressure induced by quick passaging of the virus was also associated with a multimerization of transcription factor binding sites in the LTR [24]. Here we also observed an increase in the length of the LTR (Fig. 5), but were unable to sequence the longer LTR sequences. However, multiple repeats found at the beginning of co-cultivation were in agreement with longer LTRs (Fig. 5). Soon after detection of the longer LTR variants, this virus was lost either due to a natural instability of the repeats or constraints imposed by virion packaging limits. After numerous passages, the LTR had the normal size und the correct lining up of the repeat sequences (Fig. 6). The increase of the LTR length during passaging on human cells seems to be an adaptation on rapid growth on these cells and was also described for other retroviruses. In mice and cats, lymphoma induction appeared to be dependent on the generation of viruses with duplicated enhancer sequences [40–43]. In the case of PERV these repeats are binding sites for transcription factors such as NF-Y, CEBP, HFH-3, AP-1 [24].

Using two primer pairs to amplify the *env* region helps to localize the recombination point. When the 380 bp amplicon (primer pair PERV-A VRB fw and PERV-C rev) can be detected, the breakpoint is in the region between both primers. When the 1200 bp amplicon (primer pair PERV-A VRB fw and PERV-C TMR rev) can be detected, the breakpoint is located upstream the PERV-C rev primer binding site (Fig. 1). Comparing the recombination point between different PERV-A/C isolates (see Fig. 1 and [9]), clearly demonstrates that each recombination is an independent event.

Until now, the isolation of a human-tropic PERV-A/Cs was, with one exception, only described for minipigs (for review see [9]), and our results confirm this observation. The exception were diseased farm animals [44]. The reason why minipigs release more often PERV-A/C is still unclear, a higher copy number and a higher expression of PERV-C in minipigs in comparison with other pigs is likely.

It is well known that mitogen stimulation increases the expression of PERVs in pig PBMCs [11–13]. Here we demonstrated that in mitogen-stimulated PBMCs not only the expression of PERV in general, using pol primer which recognize all PERVs (Fig. 4A), but also the expression of PERV-C (Fig. 4B) and PERV-A/C (Fig. 4C) was increased. In addition to the comparison of the expression before and after mitogen stimulation, we also compared PERV expression in the PBMCs of GöMP-F and GöMP-E with the PERV expression in PK15 cells (Fig. 4A). This allows a comparison with the PERV expression in PBMCs of other pig breeds. For example, PERV expression in mitogen-stimulated PBMCs from a Yucatan micropig was 92% of that in PK15 cells, whereas expression in Large White pigs was only 5% of that in PK15 cells [12]. In unstimulated PBMCs from hybrids between Large White, Duroc and minipigs, the expression was between 2 and 8%, in stimulated between 2 and 43% [12], The expression in unstimulated PBMCs of GöMP F was 5%, in stimulated 37% (Fig. 4A). This confirms differences in the expression of PERV in different pig breeds and in individual pigs.

## Conclusion

Our results demonstrate that the transmission of human-tropic PERV is an extremely rare event since it was found only in the case of one animal (GöMP-F) of 58 analyzed pigs. The virus was isolated and characterized in detail, it was a PERV-A/C and had longer LTR sequences due to the acquisition of additional repeats during passaging. PERV-A/C sequences were found also in PBMCs of a second GöMP (GöMP-8), however there was no virus transmission to human cells. The recombination sites of these two viruses and of all previously published PERV-A/C were different, indicating that all recombinations are independent events. To avoid PERV-A/C recombinations, only PERV-C negative animals should be used for xenotransplantation.

## Supplementary Information

**Additional file 1. Figure S1: **Sequence of a part of the envelope protein of the PERV isolated from GöMP number 8 in comparison with sequences of a PERV-A (Accession-Number AJ293656.1), a PERV-C (Accession-Number KY352351), and PERV-F [11].

## Data Availability

All data generated during this study are included in the published article and its supplementary figure.

## Abbreviations

GöMP: Göttingen minipigs
env: Envelope
LTR: Long terminal repeats
PERV: Porcine endogenous retrovirus
PBMCs: Peripheral blood mononuclear cells
RT: Reverse transcriptase
