# Supplementary material for: Rare isolation of human-tropic recombinant porcine endogenous retroviruses PERV-A/C from Göttingen minipigs
Source: Virol J. 2022 Feb 21;19:30. doi: 10.1186/s12985-022-01742-0 (PMC8862210; doi:10.1186/s12985-022-01742-0)
Supplement: Supplementary file 1 — Additional file 1. Figure S1: Sequence of a part of the envelope protein of the PERV isolated from GöMP number 8 in comparison with sequences of a PERV-A (Accession-Number AJ293656.1), a PERV-C (Accession-Number KY352351), and PERV-F [11]. [file 12985_2022_1742_MOESM1_ESM.pdf]

	1	60
PERV A (AJ293656.1):	CCTACCAGTTATAATCAATTTAATTATGGCCATGGGAGATGGAAAGATTGGCAACAGCGG	
PERV C (KY352351):	TATACCAGCTCTGGACAATTTAATTA - - - CC - - - TGACCTGGA - - - - TTAG-AACTG - - -	
PERV A/C (GöMP-F):	CCTACCAGTTATAATCAATTTAATTATGGCCATGGGAGATGGAAAGATTGGCANCAGCGG	
PERV A/C (GöMP-8):	CCTACCAGTTATAATCAATTTAATTATGGCCATGGGAGATGGAAAGATTGGCAACAGCGG	
	61	120
PERV A (AJ293656.1):	GTACAAAAAGATGTACGAAATAAGCAAATAAGCTGTCATTCGTTAGACCTAGATTACTTA	
PERV C (KY352351):	- - - - - - - - - GAAGCCC - - - - - - - - CAAGT - - GCTCTCCTTC - - - AGACCTAGATTACCTA	
PERV A/C (GöMP-F):	GTACAAAAAGATGTACGAAATAAGCAAATAAGCTGTCATTCGTTAGACCTAGATTACTTA	
PERV A/C (GöMP-8):	GTACAAAAAGATGTACGAAATAAGCAAATAAGCTGTCATTCGTTAGACCTAGATTACTTA	
	121	180
PERV A (AJ293656.1):	AAAATAAGTTTCACTGAAAAAGGAAAACAAGAAAATATTCAAAAGTGGGTAAATGGTATA	
PERV C (KY352351):	AAAATAAGTTTCACTGAGAAAGGAAAACAAGAAAATATCCTAAAATGGGTAAATGGTATG	
PERV A/C (GöMP-F):	AAAATAAGTTTCACTGAAAAAGGAAAACAAGAAAATATTCAAAAGTGGGTAAATGGTATG	
PERV A/C (GöMP-8):	AAAATAAGTTTCACTGAAAAAGGAAAACAAGAAAATATTCAAAAGTGGGTAAATGGTATG	
	181	240
PERV A (AJ293656.1):	TCTTGGGGAATAGTGTACTATGGAGGCTCTGGGAGAAAGAAAGGATCTGTTCTGACTATT	
PERV C (KY352351):	TCTTGGGGAATGGTATATTATGGAGGCTCGGGTAAACAACCAGGCTCCATTCTAACTATT	
PERV A/C (GöMP-F):	TCTTGGGGAATAGTGTACTATGGAGGCTCTGGGAGAAAGAAAGGATCTGTTCTGACTATT	
PERV A/C (GöMP-8):	TCTTGGGGAATAGTGTACTATGGAGGCTCTGGGAGAAAGAAAGGATCTGTTCTGACTATT	
	241	300
PERV A (AJ293656.1):	CGCCTCAGAATAGAAACTCAGATGGAACCTCCGGTTGCTATAGGACCAAATAAGGGTTTG	
PERV C (KY352351):	CGCCTCAAAATA -- AAC - CAGCTGGAGCCTCCAATGGCTATAGGACCAAATACGGTCTTG	
PERV A/C (GöMP-F):	CGCCTCAAAATAGAAACTCAGCTGGAGCCTCCAATGGCTATAGGACCAAATACGGTCTTG	
PERV A/C (GöMP-8):	CGCCTCAGAATAGAAACTCAGATGGAACCTCCGGTTGCTATAGGACCAAATAAGGGTTTG	
	301	360
PERV A (AJ293656.1):	GCCGAACAAGGACCTCCAATCCAAGAACAGAGGCCATCTCCTAACCCCTCTGATTACAAT	
PERV C (KY352351):	ACGGGTCAAAGACCCCCAACCCAAGGACCAGGACCAT - - - - - - - - - CCTCT - - - - - - AAC	
PERV A/C (GöMP-F):	ACGGGTCAAAGACCCCCAACCCAAGGACCAGGACCATCCTCTAACCCCTCTGATTACAAT	
PERV A/C (GöMP-8):	GCCGAACAAGGACCTCCAATCCAAGAACAGAGGCCATCTCCTAACCCCTCTGATTACAAT	
	361	420
PERV A (AJ293656.1):	ACAACCTCTGGATCAGTCCCCACTGAGCCTAACATCACTATTAAAACA - GGGGCGAAACT	
PERV C (KY352351):	ATAACTTCTGGATCAGACCCCACTGAGTCTAACAGCACGACTAAAAT - GGGGGCAAAACT	
PERV A/C (GöMP-F):	ATAWCCTCTGGATCAGTCCCCACTGAGCCTAACATCACTATTAAAACAGGGGGCGAAACT	
PERV A/C (GöMP-8):	ACAACCTCTGGATCAGTCCCCACTGAGCCTAACATCACTATTAAAACA - GGGGCGAAACT	
	421	480
PERV A (AJ293656.1):	TTTTAGCCTCATCCAGGGAGCTTTTCAAGCTCTTAACTCCACGACTCCAGAGGCTACCTC	
PERV C (KY352351):	TTTTAGCCTCATCCAGGGAGCTTTTCAAGCTCTTAACTCCACGACTCCAGAGGCTACCTC	
PERV A/C (GöMP-F):	TTTTAGCCTCATCCAGGGAGCTTTTCAAGCTCTTAACTCCACGACTCCAGAGGCTACCTC	
PERV A/C (GöMP-8):	TTTTAACCTCATCCAGGGAGCTTTTCAAGCTCTTAACTCCACGACTCCAGAGGCTACCTC	
	481	540
PERV A (AJ293656.1):	TTCTTGTTGGCTTTGCTTAGCTTCGGGCCCACCTTACTATGAGGGAATGGCTAGAGGAGG	
PERV C (KY352351):	TTCTTGTTGGCTATGCTTAGCTTTGGGCCCACCTTACTATGAAGGAATGGCTAGAAGAGG	
PERV A/C (GöMP-F):	TTCTTGTTGGCTTTGCTTAGCTTCGGGCCCACCTTACTATGAGGGAATGGCTAGAGGAGG	
PERV A/C (GöMP-8):	TTCTTGTTGGCTTTGCTTAGCTTCGGGCCCACCTTACTATGAGGGAATGGCTAGAGGAGG	
	541	600
PERV A (AJ293656.1):	GAAATTCAATGTGACAAAGGAACATAGAGACCAATGTACATGGGGATCCCAAAATAAGCT	
PERV C (KY352351):	GAAATTCAATGTGACAAAAGAACATAGAGACCAATGCACATGGGGATCCCAAAATAAGCT	
PERV A/C (GöMP-F):	GAAATTCAATGTGACAAAGGAACATAGAGACCAATGTACATGGGGATCCCAAAATAAGCT	
PERV A/C (GöMP-8):	GAAATTCAATGTGACAAAGGAACATAGAGACCAATGTACATGGGGATCCCAAAATAAGCT	
	601	660
PERV A (AJ293656.1):	TACCCTTACTGAGGTTTCTGGAAAAGGCACCTGCATAGGGATGGTTCCCCCATCCCACCA	
PERV C (KY352351):	TACCCTTACTGAGGTTTCTGGAAAAGGCACCTGCATAGGAAAGGTTCCCCCATCCCACCA	
PERV A/C (GöMP-F):	TACCCTTACTGAGGTTTCTGGAAAAGGCACCTGCATAGGAAAGGTTCCCCCATCCCACCA	
PERV A/C (GöMP-8):	TACCCTTACTGAGGTTTCTGGAAAAGGCACCTGCATAGGGATGGTTCCCCCATCCCACCA	

	661	720
PERV A (AJ293656.1):	ACACCTTTGTAACCACACTGAAGCCTTTAATCGAACCTCTGAGAGTCAATATCTGGTACC	
PERV C (KY352351):	ACACCTTTGTAACCACACTGAAGCCTTTAATCAAACCTCTGAGAGTCAATATCTGGTACC	
PERV A/C (GöMP-F):	ACACCTTTGTAACCACACTGAAGCCTTTAATCGAACCTCTGAGAGTCAGTATCTGGTACC	
PERV A/C (GöMP-8):	ACACCTTTGTAACCACACTGAAGCCTTTAATCGAACCTCTGAGAGTCAGTATCTGGTACC	

	721	780
PERV A (AJ293656.1):	TGGTTATGACAGGTGGTGGGCATGTAATACTGGATTAACCCCTTGTGTTTCCACCTTGGT	
PERV C (KY352351):	TGGTTATGACAGGTGGTGGGCATGTAATACTGGATTAACCCCTTGTGTTTCCACCTTGGT	
PERV A/C (GöMP-F):	TGGTTATGACAGGTGGTGGGCATGTAATACTGGATTAACCCCTTGTGTTTCCACCTTGGT	
PERV A/C (GöMP-8):	TGGTTATGACAGGTGGTGGGCATGTAATACTGGATTAACCCCTTGTGTTTCCACCTTGGT	

	781	840
PERV A (AJ293656.1):	TTTCAACCAAACTAAAGACTTTTGCGTTATGGTCCAAATTGTCCCCCGGGTGTACTACTA	
PERV C (KY352351):	TTTTAACCAAACTAAAGATTTTTGCATTATGGTCCAAATTGTTCCCCGAGTGTATTACTA	
PERV A/C (GöMP-F):	TTTTAACCAAACTAAAGATTTTTGCATTATGGTCCAAATTGTTCCCCGAGTGTATTACTA	
PERV A/C (GöMP-8):	TTTCAACCAAACTAAAGACTTTTGCGTTATGGTCCAAATTGTCCCCCGGGTGTACTACTA	

	841	900
PERV A (AJ293656.1):	TCCCGAAAAAGCAGTCCTTGATGAATATGACTATAGATATAATCGGCCAAAAAGAGAGCC	
PERV C (KY352351):	TCCCGAAAAAGCAATCCTTGATGAATATGACTACAGAAATCATCGACAAAAGAGAGAACC	
PERV A/C (GöMP-F):	TCCCGAAAAAGCAATCCTTGATGAATATGACTACAGAAATCATCGACAAAAGAGAGAACC	
PERV A/C (GöMP-8):	TCCCGAAAAAGCAGTCCTTGATGAATATGACTATAGATATAATCGGCCAAAAAGAGAGCC	

	901	960
PERV A (AJ293656.1):	CATATCCCTGACACTAGCTGTAATGCTCGGATTGGGAGTGGCTGCAGGCGTGGGAACAGG	
PERV C (KY352351):	CATATCTCTGACACTTGCTGTGATGCTCGGACTTGGAGTGGCAGCAGGTGTAGGAACAGG	
PERV A/C (GöMP-F):	CATATCTCTGACACTTGCTGTGATGCTCGGACTTGGAGTGGCAGCAGGTGTAGGAACAGG	
PERV A/C (GöMP-8):	CATATCCCTGACACTAGCTGTAATGCTCGGATTGGGAGTGGCTGCAGGCGTGGGAACAGG	

	961	1020
PERV A (AJ293656.1):	AACGGCTGCCCTAATCACAGGACCGCAACAGCTGGAGAAAGGACTTAGTAACCTACATCG	
PERV C (KY352351):	AACAGCTGCCCTGGTCACGGGACCACAGCAGCTAGAAACAGGACTTAGTAACCTACATCG	
PERV A/C (GöMP-F):	AACAGCTGCCCTGGTCACGGGACCACAGCAGCTAGAAACAGGACTTAGTAACCTACATCG	
PERV A/C (GöMP-8):	AACGGCTGCCCTAATCACAGGACCGCAACAGCTGGAGAAAGGACTTAGTAACCTACATCG	

	1021	1080
PERV A (AJ293656.1):	AATTGTAACGGAAGATCTCCAAGCCCTAGAAAAAATCTGTCAGTAACCTGGAGGAATCCCT	
PERV C (KY352351):	AATTGTAACAGAAGATCTCCAAGCCCTAGAAAAAATCTGTCAGTAACCTGGAGGAGTCCCT	
PERV A/C (GöMP-F):	AATTGTAACAGAAGATCTCCAAGCCCTAGAAAAAATCTGTCAGTAACCTGGAGGAATCCCT	
PERV A/C (GöMP-8):	AATTGTAACGGAAGATCTCCAAGCCCTAGAAAAAATCTGTCAGTAACCTGGAGGAATCCCT	

	1081	1140
PERV A (AJ293656.1):	AACCTCCTTATCTGAAGTGGTTCTACAGAACAGAAGGGGGTTAGATCTGTTATTTCTAAA	
PERV C (KY352351):	AACCTCCTTATCTGAGGTAGTCCTACAGAATAGAAGAGGGTTAGATTTATTATTTCTAAA	
PERV A/C (GöMP-F):	AACCTCCTTATCTGAAGTAGTCCTACAGAATAGAAGAGGGTTAGATTTATTATTTCTAAA	
PERV A/C (GöMP-8):	AACCTCCTTATCTGAAGTGGTTCTACAGAACAGAAGGGGGTTAGATCTGTTATTTCTAAA	

	1141	1200
PERV A (AJ293656.1):	AGAAGGAGGGTTATGTGTAGCCTTAAAAGAGGAATGCTGCTTTTATGTGGATCATTCAGG	
PERV C (KY352351):	AGAAGGAGGATTATGTGTAGCCTTGAAGGAGGAATGCTGTTTTTATGTGGATCATTCAGG	
PERV A/C (GöMP-F):	AGAAGGAGGATTATGTGTAGCCTTGAAGGAGGAATGCTGTTTTTATGTGGATCATTCAGG	
PERV A/C (GöMP-8):	AGAAGGAGGGTTATGTGTAGCCTTAAAAGAGGAATGCTGCTTTTATGTGGATCATTCAGG	

	1201	1260
PERV A (AJ293656.1):	AGCTATCAGGGACTCCATGAGCAAGCTCAGAGAAAGGTTAGAAAAACGTCACAAAGAAAA	
PERV C (KY352351):	GGCCATCAGAGACTCCATGAACAAGCTTAGAGAAAGGTTGGAGAAGCGTCGAAGGGAAAA	
PERV A/C (GöMP-F):	GGCCATCAGAGACTCCATGAACAAACTTAGAGAAA	
PERV A/C (GöMP-8):	AGCTATCAGGGACTCCATGAGCAAGCTCAGAGAAAGGTT**GGAGAAGCGTCGAAGGGAAAA**	

	1261
PERV A (AJ293656.1):	AGAGGCTGGCCAAGGATGGTTTGAGG
PERV C (KY352351):	GGAAACTACTCAAGGGTGGTTTGAGG
PERV A/C (GöMP-F):	
PERV A/C (GöMP-8):	**GGAAACTACTCAAGGGTGGTT - GAG -**
